# Profiling of the intestinal community of *Clostridia*: taxonomy and evolutionary analysis

**DOI:** 10.20517/mrr.2022.19

**Published:** 2023-04-20

**Authors:** Francesco Candeliere, Eliana Musmeci, Alberto Amaretti, Laura Sola, Stefano Raimondi, Maddalena Rossi

**Affiliations:** ^1^Department of Life Sciences, University of Modena and Reggio Emilia, Modena 41125, Italy.; ^2^Department of Civil, Chemical, Environmental and Material Engineering (DICAM), Alma Mater Studiorum University of Bologna, Bologna 40136, Italy.; ^3^Biogest Siteia, University of Modena and Reggio Emilia, Reggio Emilia 42124, Italy.

**Keywords:** *Clostridia*, gut microbiome, metagenomics, average amino acid identity (AAI), taxonomy, phylogenomics

## Abstract

**Aim:** *Clostridia* are relevant commensals of the human gut due to their major presence and correlations to the host. In this study, we investigated intestinal *Clostridia* of 51 healthy subjects and reconstructed their taxonomy and phylogeny. The relatively small number of intestinal *Clostridia* allowed a systematic whole genome approach based on average amino acid identity (AAI) and core genome with the aim of revising the current classification into genera and determining evolutionary relationships.

**Methods:** 51 healthy subjects’ metagenomes were retrieved from public databases. After the dataset’s validation through comparison with Human Microbiome Project (HMP) samples, the metagenomes were profiled using MetaPhlAn3 to identify the population ascribed to the class *Clostridia*. Intestinal *Clostridia* genomes were retrieved and subjected to AAI analysis and core genome identification. Phylogeny investigation was conducted with RAxML and Unweighted Pair Group Method with Arithmetic Mean (UPGMA) algorithms, and SplitsTree for split decomposition.

**Results:** 225 out of 406 bacterial taxonomic units were ascribed to *Bacillota* [*Firmicutes*], among which 124 were assigned to the class *Clostridia*. 77 out of the 124 taxonomic units were referred to a species, altogether covering 87.7% of *Clostridia* abundance. According to the lowest AAI genus boundary set at 55%, 15 putative genera encompassing more than one species (G1 to G15) were identified, while 19 species did not cluster with any other one and each appeared to belong to a diverse genus. Phylogenetic investigations highlighted that most of the species clustered into three main evolutive clades.

**Conclusion:** This study shed light on the species of *Clostridia* colonizing the gut of healthy adults and pinpointed several gaps in knowledge regarding the taxonomy and the phylogeny of *Clostridia*.

## INTRODUCTION

*Clostridium* is one of the first genera delineated in the history of bacteriology, and the first binomial name of a *Clostridium* species (*Clostridium butyricum*) dates back to 1880^[1]^. Clostridia are generally defined as spore-forming, non-sulfate-reducing obligate anaerobic bacteria with a Gram-positive cell wall. A huge number of species sharing these features have been identified over the years and classified into taxonomic groups phylogenetically related to *Clostridium*^[2]^, leading to the creation of the class *Clostridia* which has become one of the largest and most complex. Nowadays, *Clostridia* comprises six orders, among which *Clostridiales*/*Eubacteriales* include 18 families, which consist of 227 genera and hundreds of species (www.bacterio.net)^[3]^.

Members of the class Clostridia are ubiquitous bacteria that inhabit environments where oxygen is absent or depleted by other microorganisms but also can survive and disseminate in the presence of air as dormant endospores. The main habitats of *Clostridia*^[2]^ are soil, sewage, marine sediments, and the intestines of humans and animals^[4]^. They are commensals of the human gut, where they represent the major constituents of *Bacillota* [*Firmicutes*], a dominant phylum along with *Bacteroidota* [*Bacteroidetes*]^[5]^. Within intestinal *Bacillota*, the class *Clostridia* exhibits a great diversity and covers a broad metabolic and functional range, obtaining energy from different fermentative pathways that break down undigested/unabsorbed intestinal carbohydrates and proteins, yielding a variety of fermentation products, namely short-chain and branched-chain fatty acids, CO_2_, and H_2_^[6]^. They are the major intestinal producers of butyrate and propionate, generated as the end products of saccharolytic catabolism^[7]^. These short-chain fatty acids (SCFAs) are notably beneficial to human health since they improve intestinal barrier function and prevent conditional pathogens and their metabolites from crossing the barrier^[8]^. Furthermore, butyrate is an important anti-inflammatory molecule, while propionate fosters satiety and prevents hepatic lipogenesis, lowering cholesterol production^[9]^. Some *Clostridia* contribute to protein breakdown and fermentation and release harmful compounds such as ammonia and aromatics^[10,11]^. *Clostridia* are important for the development and maintenance of the immune system^[12,13]^, induce local responses, such as secretory immunoglobulin A, as well as local T helper 17 cells and regulatory T cells, and contribute to gut homeostasis^[7,14]^.

Over the years, the increase of new species of anaerobic Gram-positive bacteria ascribed to the class *Clostridia* and its descending taxonomic ranks have led to much confusion about classification and relationships among the species. Several key studies reported taxonomic reorganizations of bacterial taxa within *Clostridia*^[15]^ including the *Clostridium* genus^[16-18]^, providing deep analysis of taxonomic and phylogenetic relationships within species that share phenotypic and genetic traits. Diversities and ambiguities within *Clostridia* have been demonstrated as soon as molecular approaches became available^[15]^. With the advent of phylogenetic taxonomy^[19]^, the sequence of the gene encoding the 16S rRNA became the gold standard for taxonomic and phylogenetic analysis and supported a key reorganization of *Clostridia*^[16]^. A total of 19 clusters were identified, resulting in the description of five new genera and the proposition of eleven new species^[16]^. Subsequent studies proposed that Cluster I^[16]^ should be regarded as *Clostridium sensu stricto*; therefore, many organisms formally recognized as species of the genus *Clostridium* were transferred to new or established genera^[20]^. However, many species that retain the name “*Clostridium*” should be regarded as misclassified, being more closely related to members of other genera than to *C. butyricum*, the type species of this genus, and await reclassification^[4]^. Furthermore, in some cases, the identity of the 16S rRNA gene within the genus *Clostridium* lies below the thresholds established^[21]^.

Direct comparison of sequence data is a fast approach to getting an overview of taxonomic and phylogenetic relationships. The average nucleotide identity (ANI) of the genes shared between two genomes is now the gold standard for the delineation of bacterial species, with an ANI threshold of 95%^[22,23]^. However, for the strains presenting lower ANI values, the average amino acid identity (AAI) may be utilized to confirm the inclusion of different species in a genus, with the thresholds of 55%-60% and 85%-90%, for genus and species, respectively^[24]^. For instance, whole genome comparison strategies underpinned the reclassification of the species within the genus *Arcobacter*^[25]^ and the genera within *Methylococcales*^[26]^. Additionally, core genome and AAI are widely used to infer phyletic lines in the evolutionary history of prokaryotic species^[27]^.

The enormous number of species that comprise the class *Clostridia* prompted the research community to focus on lower-ranked taxa to resolve the evolutionary relationships and reorganize the taxonomy, with studies targeting the genera *Oscillospira*^[28]^, *Clostridium*^[29]^, and *Eubacterium*^[30]^, and the families *Christensellaceae*^[31]^, *Lachnospiraceae*^[32,33]^, *Peptostreptococcaceae*^[34]^, and *Ruminococcaceae*^[35]^.

This study aimed to profile intestinal *Clostridia* of healthy subjects and reconstruct their taxonomy and phylogeny. The relatively small number of intestinal *Clostridia* detected in human fecal samples, compared to the hundreds of known species of the class, allowed a systematic whole genome approach that confirmed the need to revise the current classification into genera. Furthermore, phylogeny investigations have been performed using AAI and core genomes, taking advantage of split decomposition analysis to check the ambiguities in the inferred phylogenesis and to evaluate consistency among the output of the diverse analysis^[36]^.

## METHODS

### Metagenomic samples

Two datasets have been used to investigate species belonging to the class *Clostridia* in fecal samples of healthy subjects: the Human Microbiome Project (HMP)^[37,38]^ and 51 publicly available metagenomes of intestinal microbiota collected from NCBI Sequence Read Archive. The 51 metagenomes were obtained from healthy subjects from four different countries, namely China (CHN), Ethiopia (ETH), United States of America (USA), and Sweden (SWE), and were deposited with the accession numbers PRJNA557323, PRJNA504891, PRJEB27308, and PRJEB7369, respectively [Supplementary Table 1]. The selected datasets were generated through whole-genome shotgun sequencing on Illumina paired-end platforms, obtaining from 9.2 × 10^6^ to 1.8 × 10^8^ reads per sample, with lengths ranging between 100 and 150 bp.

### Dataset definition and analysis

Publicly available composition profiles of 553 HMP samples analyzed with MetaPhlAn2^[39]^ were used. The 51 metagenomes retrieved from NCBI Sequence Read Archive were profiled using both MetaPhlAn2 and MetaPhlAn3^[40]^. PCoA based on Jaccard distances of the two datasets analyzed by MetaPhlAn2 was conducted with QIIME2^[41]^ by importing BIOM files. Clostridia taxonomic units were profiled in the 51 metagenomes analyzed by MetaPhlAn3 to take advantage of the updated taxonomy.

### Whole genome AAI analysis

**Figure 1:**
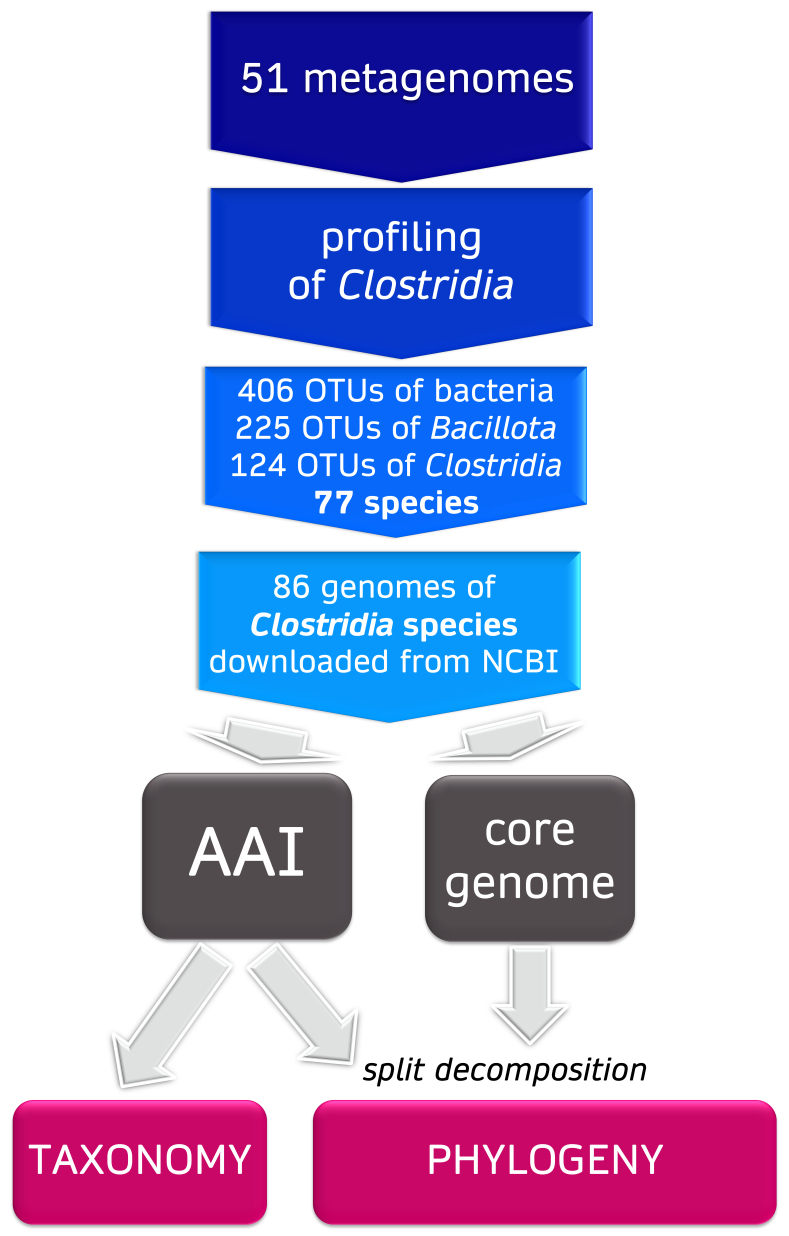
Pipeline applied to identify intestinal Clostridia, revise taxonomy, and infer phylogenetic relationships.

For each species of the class *Clostridia* identified in the 51 samples (77), the reference genome was downloaded from NCBI Datasets as of October 2021 [Figure 1]^[30,42]^. The genomes of the strains *Clostridium celatum* WC0700, *Clostridium tertium* WC 0709, and *Paraclostridium bifermentans* WC0705, previously isolated from fecal samples^[43]^, *Clostridium butyricum* DSM 10702, *C. tertium* DSM 2485, *P. bifermentans* DSM 14991, *Oscillibacter* sp. 57 20, *Eubacterium* sp. CAG180, and *Anaerotruncus* sp. CAG528 were included in the study [Supplementary Table 2].

AAI between all the genomes was calculated with the script AAI calculator of the enveomics collection (http://enve-omics.ce.gatech.edu/enveomics/) with an all-against-all approach^[44]^.

The 86 genomes of fecal *Clostridia* were annotated with Prokka^[45]^, and annotations were analyzed with Roary^[46]^ with percentage identity set at 0.5 to define the core genome. From core genome alignment, a maximum likelihood phylogenetic tree with 100 bootstrap replicates was produced using RAxML^[47]^. AAI distance matrix was used to compute the UPGMA unrooted phylogenetic tree with DendroUPGMA^[48]^ (http://genomes.urv.cat/UPGMA). The phylogenetic trees were visualized with iTOL^[49]^. The phylogeny was further inferred with SplitsTree v.4.18.2^[50]^ with a neighbor net drawing and Jukes-Cantor correction for the core genome alignment derived tree^[50,51]^.

## RESULTS

### The dataset

51 gut metagenomes of healthy subjects were analyzed with MetaPhlAn2^[39]^ and were compared with the 553 of the HMP^[37,38]^, also processed with MetaPhlAn2, to assess whether the small dataset was representative in terms of gut microbial composition. The PCoA plot of the beta diversity (computed with Jaccard dissimilarity) displayed the overall overlap of our dataset with that of the HMP, indicating the suitable representativeness of the 51 samples under investigation [Supplementary Figure 1].

**Figure 2:**
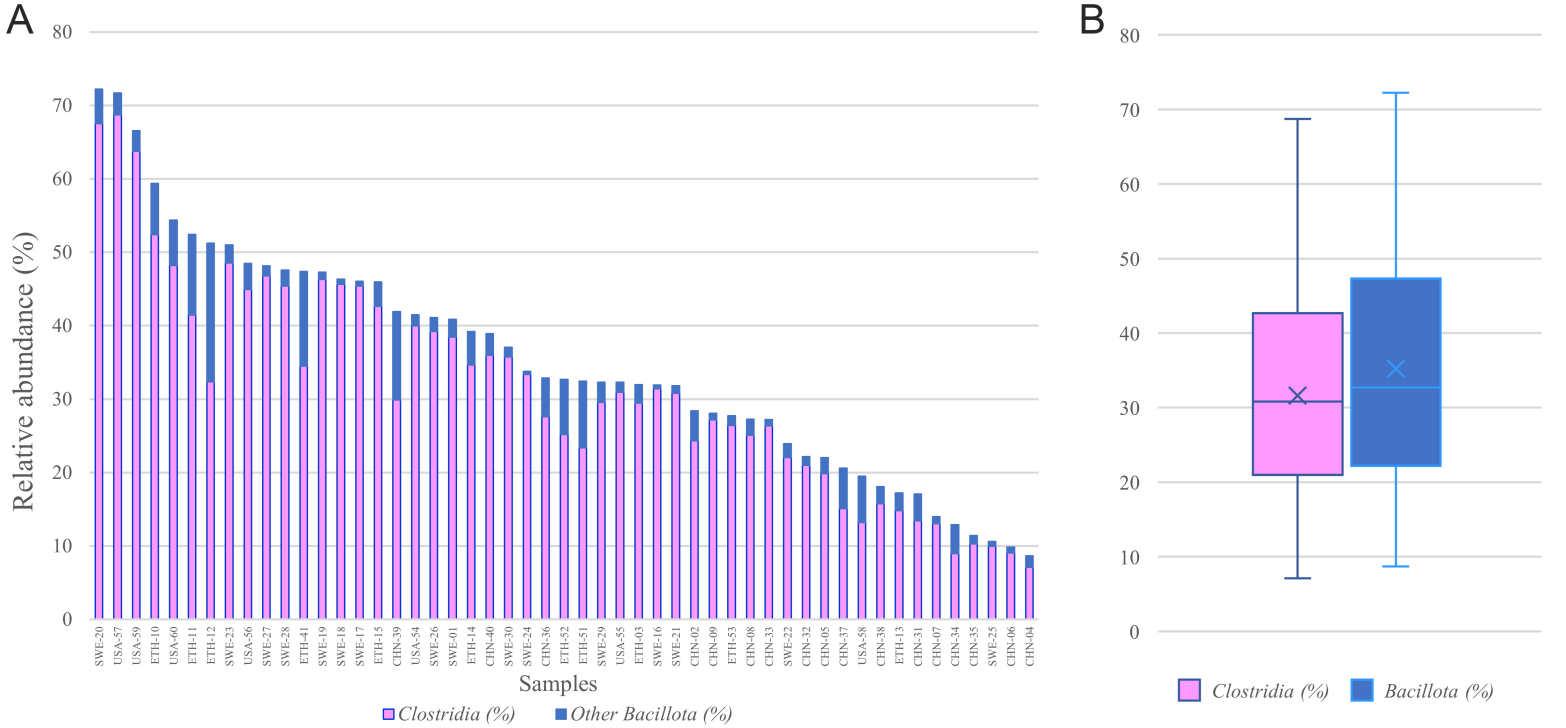
(A) Abundance in each sample of Clostridia (pink) and other Bacillota (blue); (B) boxplot of the abundance in the whole dataset of Clostridia (pink) and Bacillota (blue). Microbial composition has been assessed through analysis with MetaPhlAn3.

After validation, the smaller dataset was analyzed with MetaPhlAn3. 406 bacterial taxonomic units were identified, among which 225 were ascribed to the phylum *Bacillota*. 124 *Bacillota* taxonomic units were assigned to the class *Clostridia*, all belonging to the order *Clostridiales/Eubacteriales* [Figure 1]. *Bacillota* ranged from 8.7 to 72.2% (median 32.7%), while *Clostridia* from 7.1 to 67.5% (median 30.8%) [Figure 2A and B]. The lowest relative abundance observed in a sample for a taxonomic unit was 4 × 10^-4^%. Accordingly, a limit of detection of about 10^7^ cells g^-1^ of feces was roughly extrapolated, assuming a magnitude of microorganism concentration in the fecal samples of 10^11^ cells g^-1[52]^.

### Who and how many

77 out of the 124 taxonomic units attributed to *Clostridia* were assigned to species with recognized binomial taxonomy, altogether covering 27.7% of the whole bacterial abundance and 87.7% of *Clostridia* abundance. The remaining 47 taxonomic units, altogether accounting for a minority of the *Clostridia* population (12.3%), were not ascribed to any species. In this pool, the presence of *Oscillibacter* sp. 57 20 (mean in the 51 samples 0.8%, *n* = 46), *Eubacterium* sp. CAG180 (0.7%, *n* = 19), and *Anaerotruncus* sp. CAG528 (0.4%, *n* = 6) was relevant. The taxonomic units of *Clostridia* not identified at the species level were associated with the families *Clostridiaceae* (12), Clostridiales *incertae sedis* (2), *Eubacteriaceae* (7), *Lachnospiraceae* (10), *Oscillospiraceae* (3), and *Ruminococcaceae* (13).

**Figure 3:**
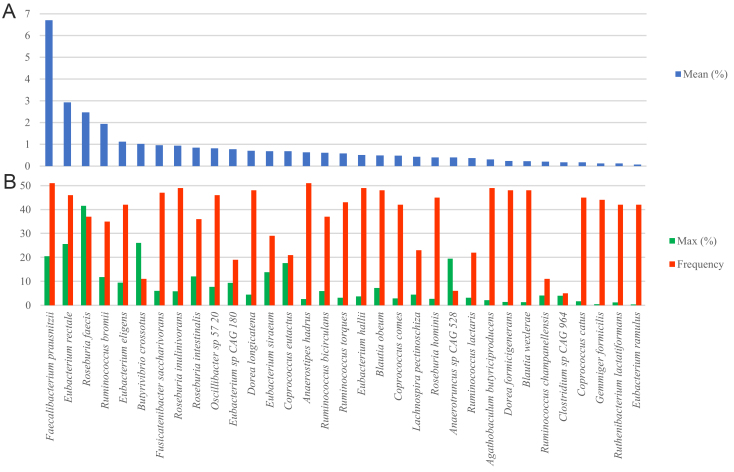
Main bacterial species of *Clostridia* detected in the fecal samples. The species reported are characterized by at least one of the following features: (A) high mean abundance (%); (B) high abundance in a single sample and frequency (N out of 51).

In general, the species that reached the highest abundance were also frequently found in the 51 microbiomes. *Faecalibacterium prausnitzii* was the most abundant and most frequently detected species. It occurred in all the 51 microbiomes, accounting on average for 6.7% and reaching up to 20.5% in sample ETH-15 [Figure 3]. Other abundant and frequently occurring species were *Roseburia faecis* (mean = 2.5%, *n* = 37); *Eubacterium rectale* [*Agathobacter rectalis*] (2.9%, *n* = 46); *Ruminococcus bromii* (1.9%, *n* = 35); *Eubacterium eligens* [*Lachnospira eligens*], *Fusicatenibacter saccharivorans*, *Roseburia inulinivorans*, *Roseburia intestinalis*, and *Dorea longicatena* (0.7% to 1.1%) [Figure 3]. Only a few species rarely detected in the microbiomes presented remarkably high abundance: *Anaerotruncus* sp. CAG528, observed only in 6 samples, reached up to 19.5% in SWE-30; *Butyrivibrio crossotus*, identified in 11 samples, reached up to 26.1% in SWE-01; *R. faecis* and *E. rectale*, each scoring in a single sample 41.5% and 25.5%, respectively.

*Anaerostipes hadrus* was detected in all the 51 samples, with a mean abundance of 0.6%. Other frequently detected species were *Eubacterium hallii* [*Anaerobutyricum hallii*] (0.5%, *n* = 49), *Agathobaculum butyriciproducens* (0.3%, *n* = 49), *Blautia obeum* (0.5%, *n* = 48), *Dorea formicigenerans* (0.2%, *n* = 48), and *Blautia wexlerae* (0.2%, *n* = 48) [Figure 3].

### Taxonomy

A set of 86 genomes of intestinal *Clostridia* were subjected to AAI comparison to validate the taxonomy and to check the genus boundaries [Figure 1 and Supplementary Table 2]. This set included 80 NCBI genomes of the clostridial detected species (i.e., the 77 species as well as *Eubacterium* sp. CAG180, *Anaerotruncus* sp. CAG528, and *Oscillibacter* sp. 57 20) and the genomes belonging to other intestinal species (i.e., *Clostridium celatum*, *Clostridium tertium*, and *Paraclostridium bifermentans*) due to their recent identification as important intestinal mucin degraders^[42]^. The genome of *Clostridium butyricum* DSM 10702^T^ was included due to its status as a type species of the genus *Clostridium*.

**Figure 4:**
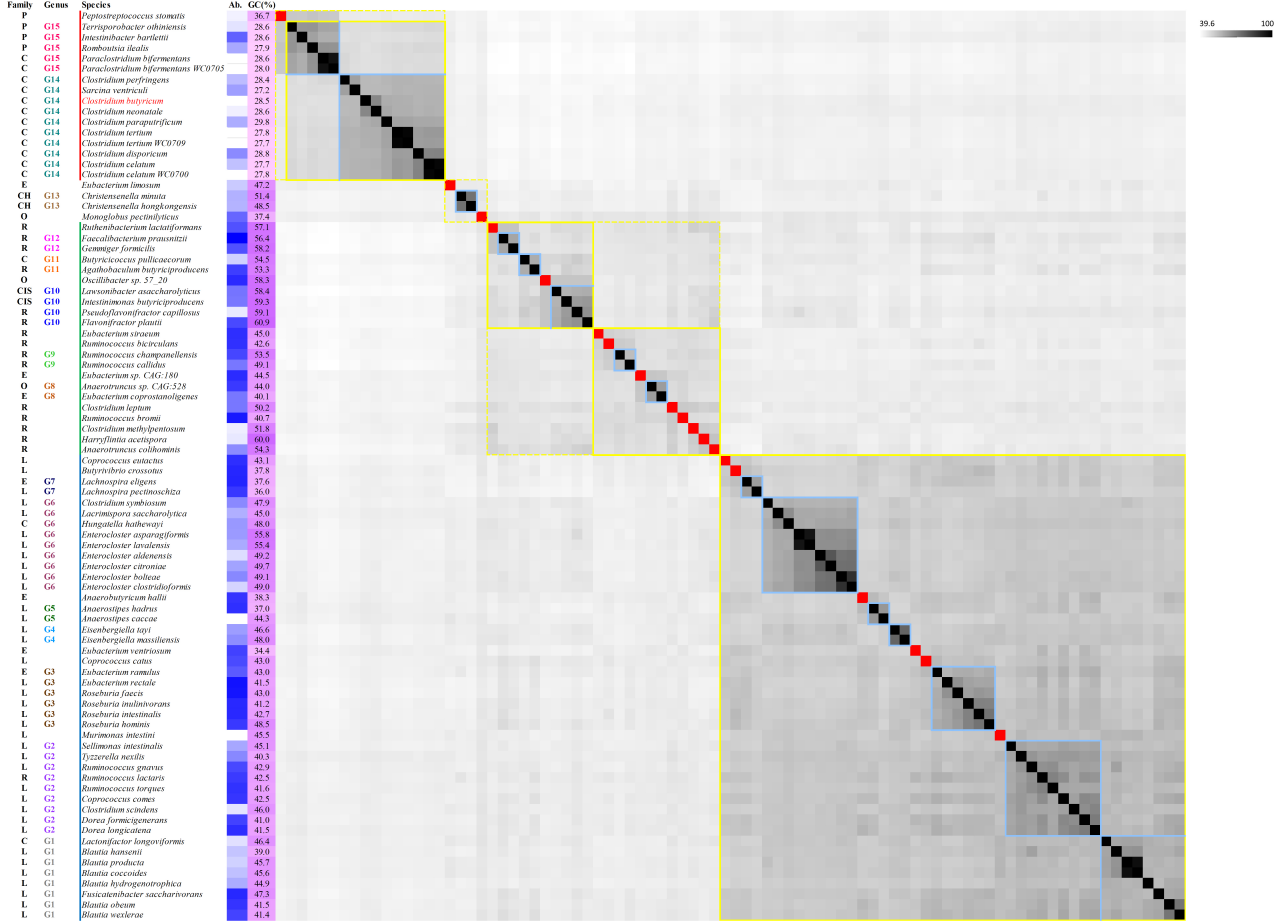
Heatmap of AAI between the 86 intestinal species of *Clostridia*, calculated with the scripts AAI calculator of the enveomics collection by all-against-all approach. The AAI values are colored following a gradient from light gray (lower values) to black (highest values). Light blue squares include predicted genera; yellow continuous or dashed line squares suggest putative families; red squares are singleton genera. Family, genus, relative abundance (Ab.), and GC% are reported. Family attribution has been retrieved from MetaPhlAn3. (*Christensenellaceae*: CH; *Clostridiaceae*: C; Clostridiales *incertae sedis*: CIS; *Eubacteriaceae*: E; *Lachnospiraceae*: L; *Oscillospiraceae*: O; *Peptostreptococcaceae*: P; *Ruminococcaceae*: R). Ab. and GC% are represented as a color gradient, from light (low) to dark blue (high) and from pink (low) to purple (high). The clusters are represented by lines (blue: Cluster 1; green: Cluster 2; red: Cluster 3).

Pairwise AAI was calculated. According to the lowest AAI genus boundary set at 55%^[24]^, 19 out of 83 species did not cluster with any other, and each appeared to belong to a different genus. Additionally, 15 putative genera encompassed more than one species (hereinafter referred to as G1 to G15) [Figure 4].

G1 included the six species of *Blautia* (*B. coccoides, B. hansenii, B. hydrogenotrophica, B. obeum, B. producta*, and *B. wexlerae*), *Fusicatenibacter saccharivorans*, and *Lactonifactor longoviformis*. They all belonged to the family *Lachnospiraceae*, except *L. longoviformis* (*Clostridiaceae*). The molar fraction of guanine and cytosine in the genomic DNA (GC%) of G1 species ranged between 39.0% and 47.3% (mean ± s.d. = 44.0% ± 2.9 %).

G2 encompassed three out of the seven species of *Ruminococcus* (*R. gnavus*, *R. lactaris*, *R. torques*), the two species of *Dorea* (*D. formicigenerans* and *D. longicatena*), *Coprococcus comes*, *Sellimonas intestinalis*, *Clostridium scindens*, and *Tyzzerella nexilis* [*Clostridium nexile*]. Except for *R. lactaris* (*Ruminococcaceae*), they all were *Lachnospiraceae*. The mean GC% of G2 was 42.6% (s.d. = 1.9%).

G3 comprised all the species of *Roseburia* (*R. faecis, R. intestini, R. inulinivorans,* and *R. hominis*), *Eubacterium ramulus*, and *E. rectale*, all assigned to the family *Lachnospiraceae*, except for *E. ramulus* (*Eubacteriaceae*). The GC% of G3 species ranged between 41.2% and 48.5% (mean ± s.d. = 44.3% ± 2.7%).

The two species of *Eisenbergiella* (*E. massiliensis* and *E. tayi*) were assigned to G4 (GC% 48.0% and 46.6%, respectively); the two species of *Anaerostipes* (*A. caccae* and *A. hadrus*) to G5 (GC% 44.3% and 37.0%, respectively). All the species clustering in G4 and G5 are currently assigned to the family *Lachnospiraceae*.

G6 encompassed the six species of *Enterocloster* (*E. aldenensis, E. asparagiformis, E. bolteae, E. citroniae, E. clostridioformis* and *E. lavalensis*), *Clostridium symbiosum*, *Hungatella hathewayi*, and *Lacrimispora saccharolytica*. The GC% of G6 species ranged between 45.0% and 55.8% (mean ± s.d. = 49.9% ± 3.5%). These species are presently assigned to the family *Lachnospiraceae*.

G7 included the two species of *Lachnospira* (*L. eligens* and *L. pectinoschiza*), both ascribed to *Lachnospiraceae*. They presented a GC% of 36.0% and 37.6%, respectively.

*Eubacterium coprostanoligenes* (GC% = 40.1%; *Eubacteriaceae*) and *Anaerotruncus* sp. CAG528 (GC% = 44.0%; *Oscillospiraceae*) were assigned to genus G8; the *Ruminococcaceae Ruminococcus callidus* and *R. champanellensis* to G9 (GC% = 49.1% and 53.5%, respectively).

*Lawsonibacter asaccharolyticus*, *Intestinimonas butyriciproducens*, *Flavonifractor plautii*, (Clostridiales *incertae sedis*) and *Pseudoflavonifractor capillosus* (*Ruminococcaceae*) were included into G10, with a GC% ranging between 58.4% and 60.9%. The species *Agathobaculum butyriciproducens* (GC% = 53.3%; *Ruminococcaceae*) and *Butyricicoccus pullicaecorum* (GC% = 54.5%; *Clostridiaceae*) were assigned to genus G11; the *Ruminococcaceae Gemmiger formicilis* and *Faecalibacterium prausnitzii* to G12, and presented a GC% of 58.2% and 56.4%, respectively.

The genus *Christensenella* (*C. hongkongensis* and *C. minuta*) of the family *Christensenellaceae* was confirmed as a separate lineage (G13). They presented a GC% of 48.5% and 51.4%, respectively.

G14 included seven species currently assigned to the genus *Clostridium* (*C. butyricum*, *C*. *perfringens*, *C*. *neonatale*, *C*. *paraputrificum*, *C*. *tertium*, *C*. *disporicum*, and *C*. *celatum*) and *Sarcina ventriculi* (*Clostridiaceae*). The GC% of G14 species ranged between 27.2% and 29.8% (mean ± s.d. = 28.2% ± 0.8%).

The group G15 included *Intestinibacter bartlettii*, *Terrisporobacter othiniensi*s, *Romboutsia ilealis* (*Peptostreptococcaceae*), and *Paraclostridium bifermentans* (*Clostridiaceae*), with a mean GC% of 28.3% (s.d. = 0.4%).

### Phylogenomics

Phylogeny of the 86 intestinal *Clostridia* was inferred by pairwise AAI calculation and core genome alignment, which consistently delineated branching into the predicted genera. In the core genome tree, most of the nodes leading to the suggested genera had a bootstrap value of 100% [Supplementary Figure 2]. Only the branches resulting in speciation of *R. bromii* and *C. leptum* (92%), *T. nexilis* and *S. intestinalis* (79%), and *I. butyriciproducens* and *F. plautii* (65%) were less reliable.

**Figure 5:**
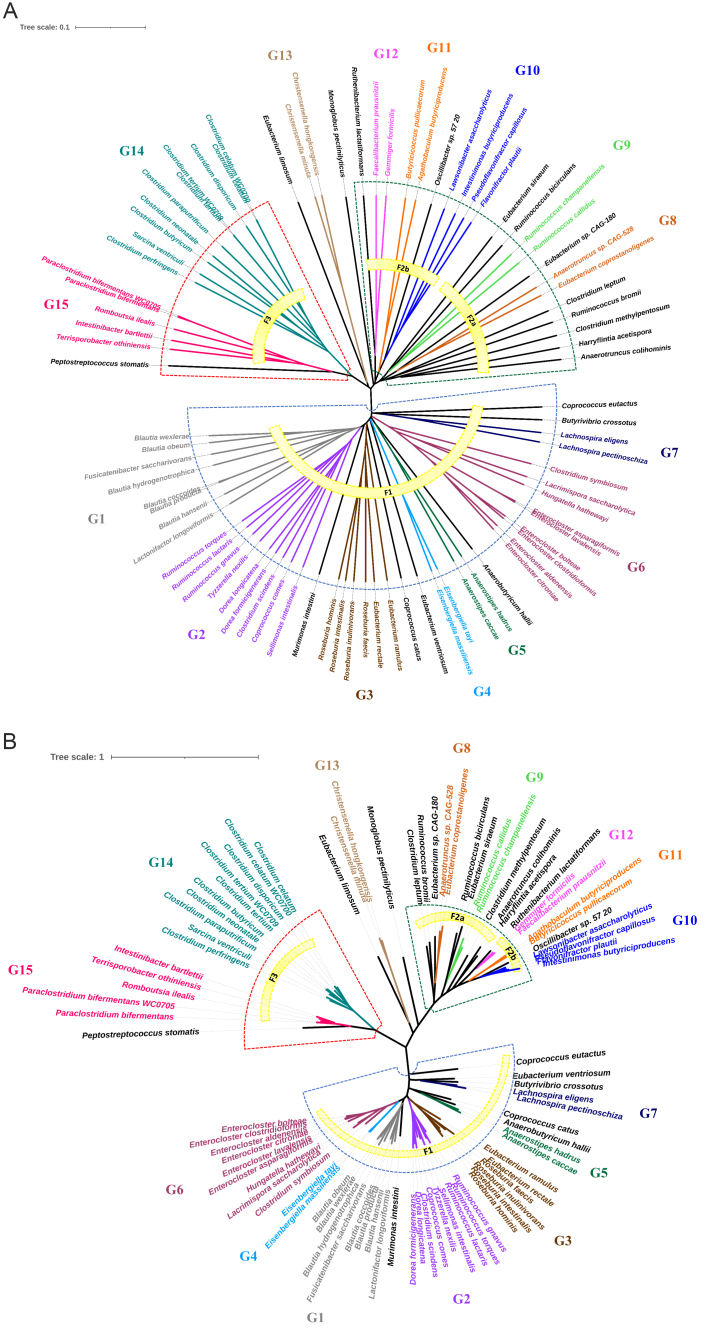
(A) Unrooted AAI and (B) core genome phylogenetic trees. Clusters, families, and putative genera are reported. Dashed lines delineate clusters C1 (blue), C2 (green) and C3 (red). AAI tree was computed with UPGMA method from AAI distance matrix, while the core genome tree was constructed with the maximum likelihood method using RAxML tool from the core genes alignment.

The topology of the phylogenetic trees consistently indicated that divergence events leading to most of the species, including those clustering into putative genera, occurred remotely [Figure 5]. Split decomposition yielded a highly reticulated network in the first events that led to the evolution from a common ancestor, with initial trajectories that could not be inferred without ambiguity [Supplementary Figure 3].

Most of the species of intestinal *Clostridia* lay in major branches and clustered in three main evolutive clades hereinafter referred to as Cluster 1, Cluster 2, and Cluster 3 (C1, C2, and C3) [Figure 5]. Only the species *E limosum, C. hongkongensis, C. minuta*, and *M. pectinilyticus* could not be assigned with certainty to C2 or C3, due to inconsistent allocation by core genome and AAI analysis, and relevant reticulation in split decomposition.

C1 encompassed 44 species grouped in 13 genera, including G1 to G7 and the singletons *M. intestini, C. catus, E. ventriosum, A. hallii, B. crossotus,* and *C. eutactus* [Figures 4 and 5]. The mean GC% of C1 species was 44.0% (s.d. = 4.6%). Considering the descendants from a common lineage and that, within C1, pairwise AAI was always > 48.5%, higher than the threshold of 45% proposed by Konstantinidis *et al.*^[53]^ for family delineation, these 13 genera could be tentatively ascribed to a single large family (F1). Within F1, stronger relationships were identified among some genera. For instance, pairwise AAI was always > 53% between species of clades G1, G2, and *M. intestini*, or between the species of G7 and *B. crossotus*. This latter association was also consistent in terms of similar GC% (mean ± s.d. = 37.1% ± 1.0%). Furthermore, within F1, some species assigned to different genera in some cases presented AAI > 55%, and more rarely > 60%. In genus G6, some clades speciated later and resulted in more closely related groupings, such as those currently assigned to the genus *Enterocloster*. AAI values and tree topology indicated that *E. asparigiformis* and *E. lavalensis* were the same species.

A solid node (bootstrap = 100%, Supplementary Figure 2) led to the strictly related species of C2, that presented a higher GC% (mean ± s.d. = 52.3% ± 6.8%) than F1 members (mean ± s.d. = 44.0% ± 4.6%). The difference in GC% content was also statistically significant according to the one-way ANOVA test (*P* < 0.05). Accordingly, with the wider range of GC%, pairwise AAI were generally lower within C2 than among genera included in F1. Strong relationships, confirmed by AAI, core genome tree topology, and GC%, were pinpointed between species of G10 and *Oscillibacter* sp. 57 20 (AAI > 53.3%), and between species of G12 and *R. lactatiformans* (AAI > 53.8%).

C3 encompassed G14, G15, and *P. stomatis* [Figures 4 and 5]. This cluster was featured by a quite low and similar GC% (mean ± s.d. = 28.8% ± 2.3%). The AAI between G15 and *P. stomatis* was > 52.7%. The genus G14 and the clade with the closely related G15 and *P. stomatis* remotely diverged [Figure 5]. The species of G14 and G15 also presented strong associations (> 46.9%) and similar GC content (mean 28.7% ± s.d. 0.7%), while the AAI of *P. stomatis* against species of G14 was in the range of 44.5%-45.2%. In terms of GC%, *P. stomatis* was an outlier within C3 (GC% 36.7%).

Unlike C1, which coincided with a single family (F1), C2 encompassed two putative separate families, namely F2a (including G8, G9, and a number of singleton species; Figure 4) and F2b (including G10, G11, G12, *Oscillibacter* sp. 57 20, and *R. lactatiformans*). The putative families F2a and F2b presented different GC% mean values (57.5% ± 2.3% and 48.0 ± 6.2%, respectively).

AAI values within C3 did not allow a clear delineation of families consistent with the 45% AAI threshold. *E. limosum, M. pectinilyticus*, and G13 (*C. hongkongensis* and *C. minuta*) remained excluded from the three main clusters. AAI between *E. limosum* and *M. pectinilyticus*, *C. hongkongensis,* and *C. minuta* ranged between 44.4% and 44.7%, slightly under the 45% limit proposed, while *M. pectinilyticus* AAI against *E. limosum, C. hongkongensis*, and *C. minuta* showed higher values (44.4%-46.0%). Then again, *M. pectinilyticus* presented a high genetic relationship also with species of F2b, with a mean AAI of 45.4% and a minimum of 43.8%.

## DISCUSSION

The class *Clostridia* includes a large and diverse number of taxa whose classification is often problematic. This study focused on the identification and quantification of *Clostridia* inhabiting the human gut of healthy subjects. It attempted a taxonomic reorganization of intestinal species and improved the current understanding of the phylogeny and interrelationships of the members of the class.

Analysis of WGS metagenomes of 51 fecal samples from healthy adults furnished some indicative information about relative abundances bypassing the amplification step, which can cause a further bias on abundance estimates. This approach pinpointed only the most abundant taxa (approx. > 10^7^ cell g^-1^) and neglected the sub-dominant population that may inhibit the gut. The absolute abundance of intestinal *Clostridia* was skewed since the community size can vary among individuals^[54]^.

Despite the low number of samples present in the dataset, the 51 metagenomes under observation included hosts of different geographical origins^[55]^ with diverse genetic, nutritional, and lifestyle features, and properly overlapped the diversity represented by the 553 metagenomes of the HMP.

80 taxa, corresponding to 77 species and 3 taxonomic units not classified at the level of species, represented 94.2% of the whole abundance of *Clostridia*, while the other 44 taxonomic units covered the remaining 5.8%, representing a sub-dominant population that could nonetheless interact with the host and exert some relevant role. For instance, *Clostridioides difficile* was not detected in any sample, according to the good health status of the subjects and the identification of this species only in 5 out of 553 samples of the HMP dataset (data not shown).

Before molecular methods were available, microbiologists relied on laboratory isolation and enumeration to survey bacterial communities, strategies that give valuable information on microbial localization, abundance, and physiology, but that generally offer a skewed and partial picture of community diversity and composition. For instance, *C. butyricum*, claimed as one of the most frequently isolated clostridial species from fecal samples^[56]^, was not detected in any of the 51 samples observed in this study nor in the 553 HMP metagenomes. However, the assignment of taxonomic labels to metagenome sequences with Kraken2^[57]^ identified *C. butyricum* in all the51 samples, although in negligible amount (mean = 0.004%).

Only *F. prausnitzii* and *A. hadrus* were found in all the samples, the former in higher amounts. These species are butyrate-producing saccharolytic gut commensals positively associated with host health and present a decreased population in patients with inflammatory bowel diseases, irritable bowel syndrome, and metabolic disorders^[58]^. According to the AAI threshold, *F. prausnitzii* was included in the putative genus G12 of C2 with *Gemmiger formicilis*, another butyrate-producer with anti-inflammatory activity^[59]^. *A. hadrus* is another saccharolytic commensal that produces butyrate from carbohydrate metabolism and, mainly for this property, is positively associated with host health^[60]^. *A. hadrus* belonged to the genus G5 of C1 with *A. caccae*, the latter resulting a promising biomarker in early screening of colorectal cancer^[61]^.

*Roseburia* species (*R. faecis, R. hominis, R. intestinalis, R. inulinivorans*) are commensals that produce SCFA from carbohydrate fermentation. They all clustered into the genus G3 of C1, together with *E. ramulus* and *E. rectale*, the latter detected in most of the samples (46/51). *R. faecis* and *E. rectale* reached remarkably high levels in a few metagenomes, although their relevance in the human microbiome has already been discussed by Karcher *et al.*^[62]^. The phylogenetic relationship associating *E. ramulus, E. rectale,* and *Roseburia* spp. was already identified by the alignment of 16 ribosomal marker proteins in a study aimed at revising the phylogenesis of the *Eubacterium* species and exploring their effects on host health^[30]^. Interestingly, *E. ramulus* and *E. rectale* are also saccharolytic butyrate producers.

Other dominant butyrate-producing *Clostridia* are currently ascribed to the genus *Eubacterium* and may ultimately be considered beneficial to human health. Among them, *E. ventriosum* formed a singleton genus of the putative family F1, *E. coprostanoligenes*, *E. sireum*, and *Eubacterium* sp. CAG180 were singletons of C2, and *E. limosum* was a stand-alone singleton outside any cluster. The current taxonomy of the genus *Eubacterium* clearly suffers from major inconsistencies that need to be resolved.

The genus *Ruminococcus* is problematic since some species (*R. torques, R. gnavus, and R. lactaris*) belong to G2 within C1, while others belong to C2. Ruminococci within G2 included frequently encountered species, such as *R. torques*. With regards to the ruminococci belonging to C2, *R. callidus* and *R. champanellensis* clustered in genus G9, closely associated with the singleton “*R. bicirculans*” (AAI > 51), and at a lower extent to *E. sireum* (AAI > 49). These relationships were also confirmed by core genome comparison. These *Clostridia* lying within C2 share the SCFA production and the promotion of a healthier status^[63]^. *R. callidus* produces SCFA and is considered a biomarker for improving health^[64]^, and *R. champanellensis* exerts a main role in cellulose degradation in the gut^[65]^. The species *R. bromii* derived from a diverse lineage within C2, even though its origin could not be easily deciphered, according to the split decomposition of phylogenetic trees.

Abundances of the clostridial species involved in mucin degradation *C. celatum*, *C. tertium*, and *P. Bifermentans* were negligible. These bacteria are expected to be components of the mucus-associated microbiota, then less represented in fecal samples. Genome-based phylogeny indicated that the genera G14 (*C. celatum* and *C. tertium*) and G15 (*Paraclostridium bifermentans*) were closely related and, together with *P. stomatis*, formed the cluster C3. G14 includes the pathogens *C. perfringens* and *C. neonatale* ^[66]^, and *C. butyricum*, the type species of the genus^[16]^ previously ascribed to the Cluster I of the *Clostridium* genus *sensu stricto*^[20,67]^.

According to our results, the dominant species of *Clostridia* in the fecal samples of healthy subjects are saccharolytic and produce SCFA. Moreover, the *Clostridia* identified as protein or mucin degraders were not detected and are likely part of a minor bacterial population, always below the limit of detection. Accordingly, proteolytic species of *Dorea*, *E. oxidoreducens*, and *Lachnoclostridium*^[10,11]^ were not detected. In healthy subjects, gut-dwelling *Clostridia* clearly expand the ecological capabilities of the host, providing a vast array of pathways to ferment complex carbohydrates and to produce organic SCFA, with an expected contribution also in terms of synthesis of limiting nutrients such as essential amino acids and vitamins^[7]^.

Profiling human intestinal *Clostridia* confirmed that this population is dominated by bacterial lineages that are never or rarely detected outside the hosts, proving their presence as commensal evolved specifically within the gut microbiome. However, the presence of a transient population acquired from food or other environmental sources is still possible, albeit it was likely not detected in this study because of lower titres.

The phylogenetic trees produced in this study all supported the existence of three distinct monophyletic groups of genera and species that accommodate most intestinal *Clostridia*. With the exception of *E. limosum*, *C. hongkongensis*, *C. minuta*, and *M. pectinilyticus*, core genome, AAI phylogeny, and GC% consistently clustered most of the genera and species into the three clusters C1, C2, and C3. This likely reflects the dominance in the gut of microorganisms highly specialized to inhabit this niche, which evolved with host lineages over evolutionary time^[68]^. The species of C3, including the genera previously ascribed to Cluster I^[16,20]^, were much less abundant than those of C1 and C2 [Figure 4].

A distribution of genera and species of the class *Clostridia* into family-level taxa can provide a more coherent taxonomic framework that reflects the phylogenetic relationships. With the AAI threshold of 45%, the main cluster C1 corresponded to a family and C2 included two families, namely F2a and F2b. An AAI threshold of 43.8% would unify F2a and F2b in a single family, coinciding with the cluster itself and also including *M. pectinilyticus*. Likewise, C3 can be considered a unique family if the AAI threshold is lowered from 45% to 44.5%, also including *P. stomatis*. A threshold of 44.5% would also group *E. limosum, C. hongkongensis, C. minuta* into a new family.

The three main clusters identified in this study are consistent with the classification of clostridial species first proposed by Collins *et al.*^[16]^. In particular, C1 matches the cluster XIVa, C2 the IVa, and C3 the I *Clostridium sensu stricto*. All the species currently assigned to *Lachnospiraceae*, with some others of *Clostridiaceae*, *Eubacteriaceae*, and *Ruminococcaceae* are located in C1 [Figure 4]. C2 encompasses the majority of *Ruminococcaceae*, all the species currently belonging to *Oscillospiraceae*, Clostridiales *incertae sedis*, *Christensellaceae*, and some species of *Eubacteriaceae* and *Clostridiaceae*. Similarly, most of the *Clostridiaceae* and all the *Peptostreptococcaceae* are included in C3. As a whole, the outcome of this study is generally consistent with both the phylogenesis and taxonomy assigned by the GTDB database (data not shown).

This study shed light on the species of *Clostridia* colonizing the gut of healthy adults and pinpointed several gaps in knowledge regarding the taxonomy and the phylogeny of *Clostridia*. The need to proceed toward a reclassification of *Clostridia* is not new. The relatively low number of species of *Clostridia* detected in the 51 metagenomes allowed us to disentangle the evolutionary relationships and establish a whole-genome-based phylogeny for the currently available genome sequences of intestinal *Clostridia*. The outcome of this study provides relevant information that can be useful to systematically resolve genus-level taxonomic inconsistencies within the whole set of *Clostridia*, regardless of the isolation niches.

## DECLARATIONS

### Availability of data and materials

The authors confirm that the metagenomes used for this work are publicly available, and their accession numbers are listed in the supplementary material [Supplementary Table 1].

### Copyright

© The Author(s) 2023.
