# Supplementary material for: Profiling of the intestinal community of Clostridia: taxonomy and evolutionary analysis
Source: Microbiome Res Rep. 2023 Apr 20;2(2):13. doi: 10.20517/mrr.2022.19 (PMC10688793; doi:10.20517/mrr.2022.19)

Supplementary Figure 2: core genome alignment tree reporting bootstrap values. Branch length is ignored for clarity.

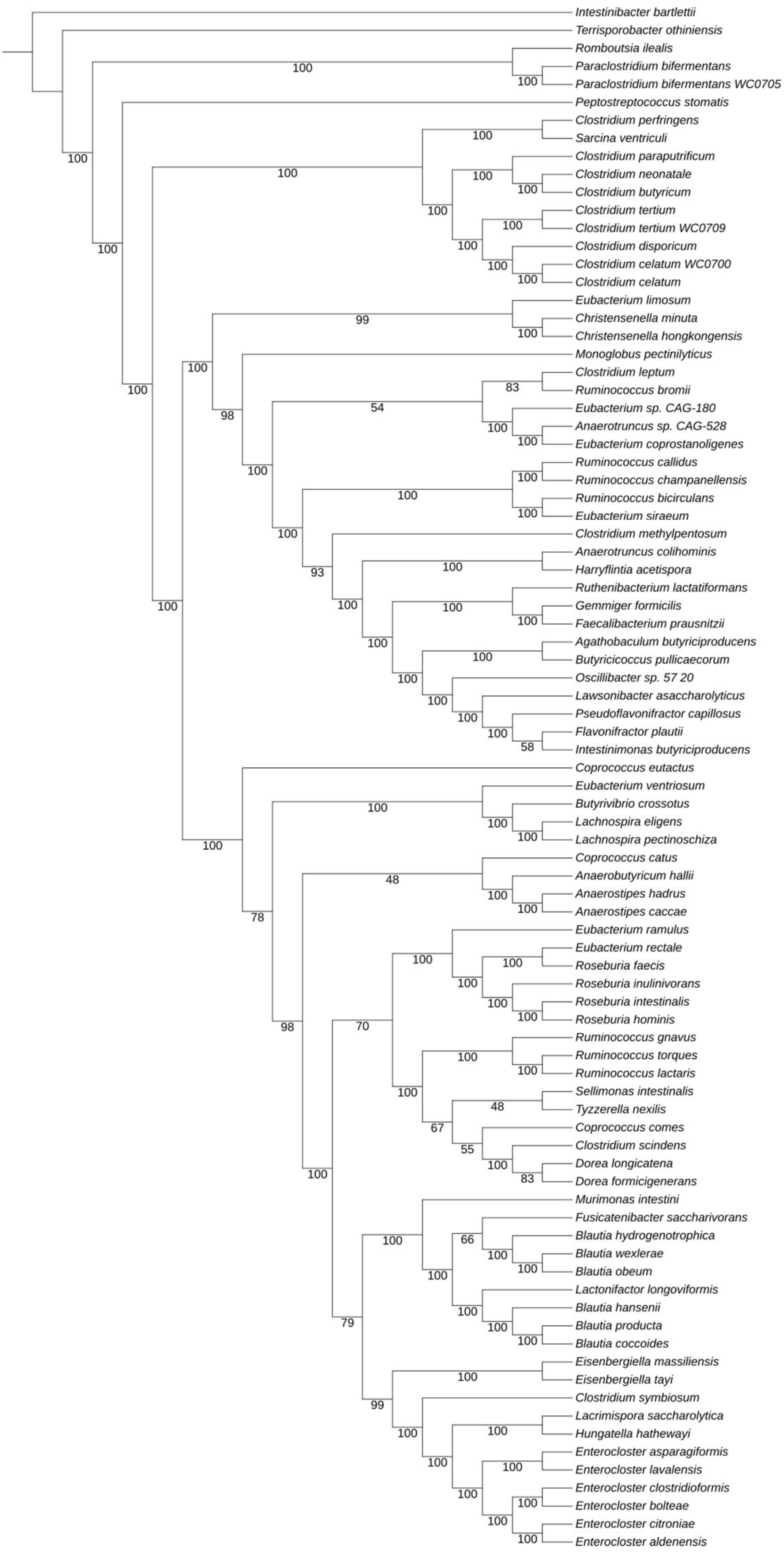

Supplement: Supplementary file 1 [file mrr-2-2-13-SupplementaryMaterials.zip › Supplementary Figure 2.pdf]
