# Supplementary material for: Profiling of the intestinal community of Clostridia: taxonomy and evolutionary analysis
Source: Microbiome Res Rep. 2023 Apr 20;2(2):13. doi: 10.20517/mrr.2022.19 (PMC10688793; doi:10.20517/mrr.2022.19)

Supplementary Figure 3: Split decomposition of phylogenetic trees base on core genes alignment (A) and AAI (B). The analysis was performed utilizing gene alignments for core genome genes and distance matrix for AAI.

A

B

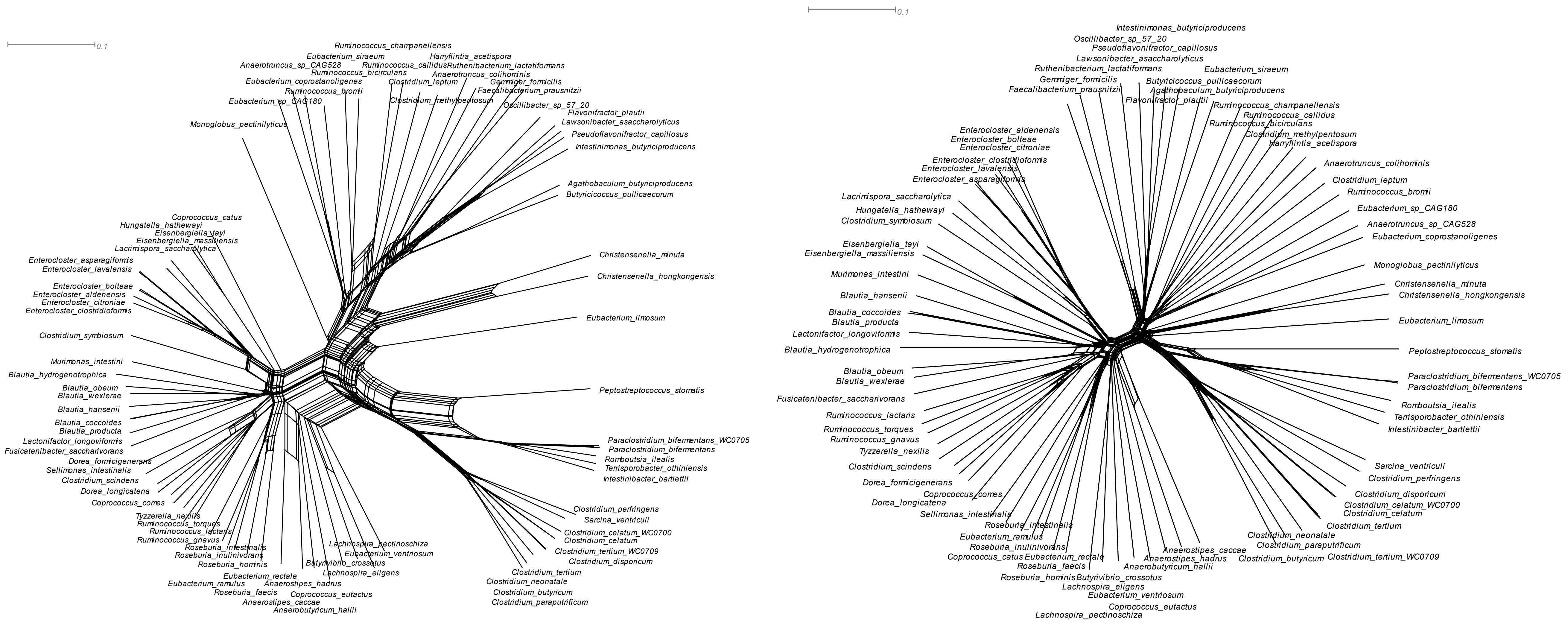

Supplement: Supplementary file 1 [file mrr-2-2-13-SupplementaryMaterials.zip › Supplementary Figure 3 .pdf]
