# Supplementary material for: Profiling of the intestinal community of Clostridia: taxonomy and evolutionary analysis
Source: Microbiome Res Rep. 2023 Apr 20;2(2):13. doi: 10.20517/mrr.2022.19 (PMC10688793; doi:10.20517/mrr.2022.19)

Supplementary Figure 1: PCoA plot of beta diversity based on Jaccard dissimilarity index between 553 HMP samples and 51 samples considered in this work. The bacterial composition of both datasets was assessed with MetaPhLan2.

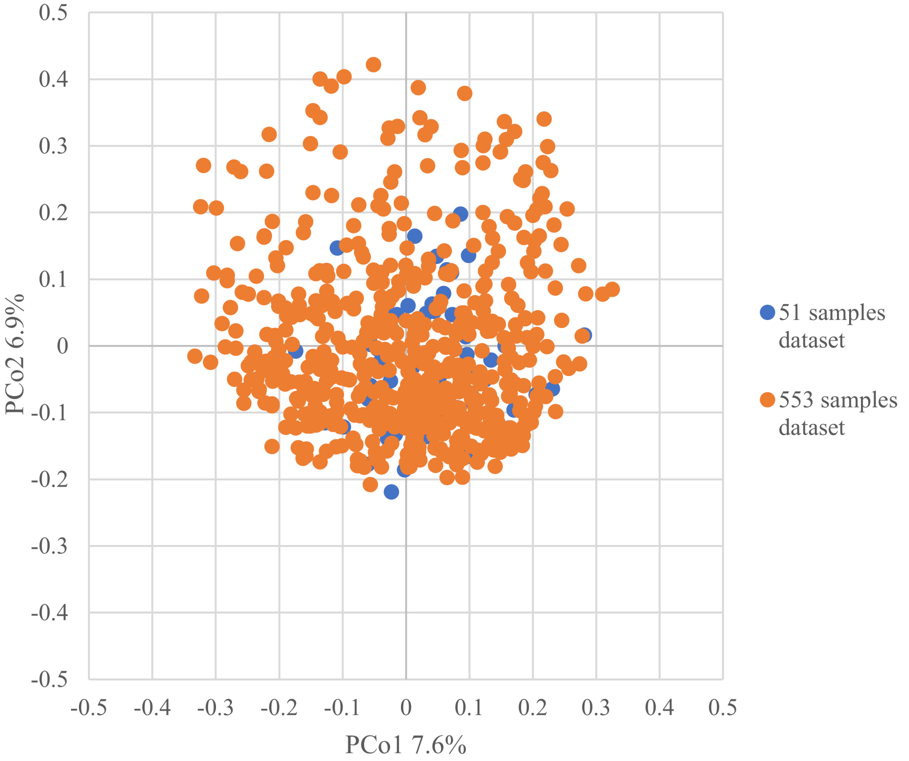

Supplement: Supplementary file 1 [file mrr-2-2-13-SupplementaryMaterials.zip › Supplementary Figure 1.pdf]
